# Fast Parabola Detection Using Estimation of Distribution Algorithms

**DOI:** 10.1155/2017/6494390

**Published:** 2017-02-21

**Authors:** Jose de Jesus Guerrero-Turrubiates, Ivan Cruz-Aceves, Sergio Ledesma, Juan Manuel Sierra-Hernandez, Jonas Velasco, Juan Gabriel Avina-Cervantes, Maria Susana Avila-Garcia, Horacio Rostro-Gonzalez, Roberto Rojas-Laguna

**Affiliations:** ^1^Division de Ingenierias, Campus Irapuato-Salamanca (DICIS), Universidad de Guanajuato, Carr. Salamanca-Valle Km 3.5+1.8, Palo Blanco, 36885 Salamanca, GTO, Mexico; ^2^CONACYT, Centro de Investigacion en Matematicas (CIMAT), A.C., Jalisco S/N, Col. Valenciana, 36000 Guanajuato, GTO, Mexico; ^3^CONACYT, Centro de Investigacion en Matematicas (CIMAT), A.C., Fray Bartolome de las Casas 314, Barrio La Estacion, 20259 Aguascalientes, AGS, Mexico

## Abstract

This paper presents a new method based on Estimation of Distribution Algorithms (EDAs) to detect parabolic shapes in synthetic and medical images. The method computes a virtual parabola using three random boundary pixels to calculate the constant values of the generic parabola equation. The resulting parabola is evaluated by matching it with the parabolic shape in the input image by using the Hadamard product as fitness function. This proposed method is evaluated in terms of computational time and compared with two implementations of the generalized Hough transform and RANSAC method for parabola detection. Experimental results show that the proposed method outperforms the comparative methods in terms of execution time about 93.61% on synthetic images and 89% on retinal fundus and human plantar arch images. In addition, experimental results have also shown that the proposed method can be highly suitable for different medical applications.

## 1. Introduction

In the pattern recognition field, detection of curves in natural or medical images is a significant and challenging problem since relevant information about an object is highly related to the shape of its boundary. Any curve can be detected by using the Hough transform (HT), if this curve can be represented by a parametric equation [1, 2].

Circular Hough transform (CHT) is based on the Hough transform principle and it has been adapted for the detection of circles [3]. For this particular problem, three different parameters (*x*_0_, *y*_0_, *r*) that define a circle have to be determined. The parameters (*x*_0_, *y*_0_) represent the coordinate of the center and *r* represents the radius [4]. Although the CHT is able to detect suitable approximation to circles in different type of images, the computational time to detect a single curve is high. This disadvantage is due to the fact that each single pixel represents a potential center (*x*_0_, *y*_0_), and a range of possible radii have to be tested for each particular pixel. On the other hand, the voting accumulator [5] that indicates the parameters of a curve that exists in the image is computed on a matrix where each cell has the number of intersections of the circles formed by taking a single pixel as the center; if the matrix is plotted in a three-dimensional graph, sharp peaks will be visualized in the cells that have more intersections. The parameters of the circle are computed by taking the highest peak in the accumulator. Since the representation of an accumulator is a matrix, two of the three parameters of the circle are represented by the rows and columns; in order to cover the third parameter, a three-dimensional matrix is needed. Although it is possible that several sharp peaks will be formed in the accumulator, when the radius of the tested circle is close to the radius of the circle, these peaks do not represent a real circle. Therefore, once the accumulator has been computed, the next challenge is to find the peak that actually represents the real circle. Due to the nature of the CHT, it would be virtually impossible to find multiple circles in an image; therefore, some studies have proposed different techniques to solve this issue by using techniques such as genetic algorithms [6] or Harmony Search (HSA) [7]. Ayala-Ramirez et al. [6] optimize the computational time of the CHT, by applying a strategy consisting in selecting three random pixels from the edge image as the chromosomes for the genetic algorithm. These three points are used to calculate a center and radius of a circle, and the fitness function evaluates how many pixels of a virtual circle are actually present in the real edge image.

The problem of detecting parabolas can be accomplished by finding the basic parameters of the general equation for parabolic shapes, which are the vertex in (*x*_0_, *y*_0_), the angle, and its aperture. In literature, different methods have been proposed to detect parabolas using the Hough transform as a baseline. Maalmi et al. [8] proposed a method that applies genetic algorithms to perform the voting process of the Hough transform. The detected curves are horizontal and the aperture is fixed. This implementation was reported for the detection of crack defects in B-scan images. Oloumi and Rangayyan [9] applied the Hough transform for parabola detection using three parameters: (*x*_0_, *y*_0_) that represent the vertex and *a* to describe the latus rectum or aperture. The main application reported of this method is the detection of temporal arcade in retinal fundus images, where parabolas fit retina vessels [10, 11]. Other reported works also have been focused on the human eye, for finding the iris [12], and in retina angiography [13].

Another potential application for parabola detection can be seen in medical images for orthopedic diagnostics in the plantar arch. Some of the basic human movements are walking and running; these movements are possible thanks to a complete set of muscles working together. However, if the plantar arch of the human feet is not of the correct size, a set of problems (e.g., back problems) can lead to surgery and prosthetics [14]. In patients with diabetes, it was observed that there is a relationship between forefoot and rear foot pressure with the ulceration on the human foot [15]. Different methods have been proposed to address problems in the orthopedic area. P. S. Kulkarni and V. B. Kulkarni [16] proposed a method to classify the human footprint using a parameter that they call Footprint Index (FPI). First, they found the minimum distance from *y*-axis on the lateral upper and lower part of the footprint; with those two points, they draw a line *C*, where the middle point of *C* will be the center of the arch. Considering this center, two lines *B* and *A*, which are perpendicular to *C*, are computed. These lines will provide the distances from *C* to the arch vertex and from the arch vertex to the edge foot, respectively. The FPI parameter is calculated as the ratio of *B* to *A*. Then, the footprint can be classified as flat foot, normal foot, or high arch foot. Chu et al. [17] proposed a similar process as in [16]; however, some other measurements were computed and were added to the ratios proposed by P. S. Kulkarni and V. B. Kulkarni [16] in an effort to compute the arch height. Zheng et al. [18] used the footprint to perform an analysis and match a subject's gait with the corresponding footprint.

Some other approaches have been proposed for parabola detection. Salehin et al. [19] proposed conic detection by applying Pascal's theorem (i.e., approximating the curve from two tangent lines and a point from the conic). Merazi-Meksen et al. [20] detected parabolic forms from Time-Of-Flight Diffraction images in order to analyze material defects. Detection procedure is named randomized Hough transform and is a combination of Least Squares, Randomized Sampled Consensus (RANSAC), and Hough transform. Certainly, robust fitting may be seen as a nonlinear optimization problem that could be solved iteratively by RANSAC method; in particular, this method is able to cope with outliers to estimate the parameter of a desired mathematical method. RANSAC is a simple and powerful method that could be useful in many applications; depending on the model it could have many parameters to tune, but the probability of convergence increases as more iterations are used. The convergence is not warranted because initialization is chosen randomly from a small data subset (i.e., results are not repeatable). In some special cases, RANSAC is not always capable of obtaining the optimal results for well-conditioned data [21, 22].

Most state-of-the-art algorithms use Hough transform for model fitting which is very time demanding. EDAs represent a stochastic optimization technique similar to genetic algorithms, which has begun to attract more attention for solving different problems in the area of image analysis [23, 24]. One of the main advantages of EDAs (UMDA) is that they use global statistical information of the best solutions instead of a crossover or mutation operators. On the other hand, UMDA has only two parameters to be tuned, number of individuals and selection rate, since the number of generations can be replaced by another convergence criterion such as the average or standard deviation of the population.

In this paper, a new method for the parabola detection problem based on Estimation of Distribution Algorithms (EDAs) is proposed. The method is evaluated in terms of computational time on synthetic and medical images of the retina and human plantar arch. Since EDAs represent an evolutionary computation technique, the fitness function used in this work is based on the Hadamard product. EDAs have shown remarkable advantages in order to solve optimization and model fitting problems. In our proposed approach, Univariate Marginal Distribution Algorithm (UMDA) [23] performs a single detection of a parabola 94% faster than HT and 45% faster than RANSAC.

Finally, the results of the proposed method are compared with those obtained by using the Hough transform implementation of Sanchez found in the MATLAB® central [25], the parabolic shapes detection provided by the MIPAV® [26] software, and additionally the well-known RANSAC method proposed by Fischler and Bolles [27]. The remainder of this paper is organized as follows: in Section 2, the fundamentals of the Hough transform, the Estimation of Distribution Algorithms, and the proposed method for detecting parabolic shapes are explained in detail. Experimental results are presented and discussed in Section 3, and conclusions are given in Section 4.

## 2. Methods

### 2.1. Parabola Detection

The detection of curves can be achieved by exploring the duality between points on a curve and the parameters representing that curve; this method is known as Hough transform (HT) [28]. Geometric curves such as circles, ellipses, parabolas, and hyperbolas can be parameterized in a polar coordinate system (*r*, *β*), if the equation that represents the curve satisfies one of the following two equations:

(1) r=de1±ecos⁡β,r=de1±esin⁡β,

where *e* represents the eccentricity and *d* is the shortest distance between the focal points and the directrix of the curve. Since the present work is focused on the detection of parabolic curves, in these equations, the *e* parameter must have a value of 1. The most common parameters to identify a parabola are the vertex (*x*_0_, *y*_0_), the coordinates (*f*_*x*_, *f*_*y*_) of the focus, the orientation *θ*_0_ of the axis of symmetry with respect to the coordinates axes, and the coefficients of (1).

**Figure 1:**
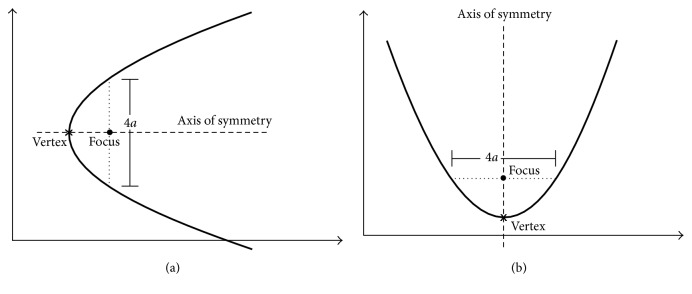
Parametric curves described by (2) and (3), respectively.

On the other hand, the equation in the Euclidean space that represents a parabolic curve with directrix parallel to the *y*-axis can be defined as follows:

(2) $$\begin{array}{c} {\left(\;y-y_{0}\;\right)}^{2}=4a\left(\;x-x_{0}\;\right), \end{array}$$

and if the directrix is parallel to the *x*-axis, the equation is modified as follows:

(3) $$\begin{array}{c} {\left(\;x-x_{0}\;\right)}^{2}=4a\left(\;y-y_{0}\;\right), \end{array}$$

where the parameter *a* is used to modify a parabola in two different aspects: the aperture direction and its magnitude. For large values of |*a*|, the aperture is increased, and if *a* is positive, the parabola of (2) opens up or to the right, while in (3) the parabola opens to the positive *y*-axis, as it is shown in Figures 1(a) and 1(b), respectively. Hence, a parabolic shape is completely determined in the Euclidean space by defining the set of parameters {*x*_0_, *y*_0_, *a*}.

**Figure 2:**
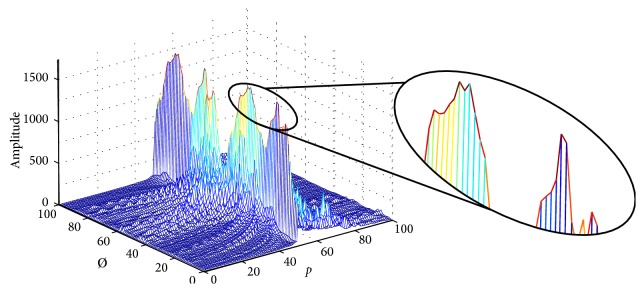
Example of a parabola accumulator using the Hough transform.

To detect parabolic shapes in images using the Hough transform algorithm, all the pixels with intensity different to zero and with coordinates (*x*, *y*) represent a potential curve in the Hough space. However, a drawback of the Hough transform is the resolution used to generate the accumulator because the input parameters such as the aperture are unknown. Consequently, there is a tradeoff between precision and fast execution time. When the tested aperture is close to the real aperture, the accumulator shows several peaks with high magnitude as it can be seen in Figure 2, and by using a low precision for the aperture parameter, the real parabola may not be found.

The main disadvantages of the HT are the computational time it takes to determine the best parameter values and the selection of the optimal peak in the accumulator, where the most commonly strategy used to find it is the local maxima method.

### 2.2. Estimation of Distribution Algorithms

The Estimation of Distribution Algorithms (EDAs) represent an extension to the field of evolutionary computation (EC). EDAs are useful to solve problems in the discrete and continuous domain by using some statistical information of potential solutions, also called individuals [29–31]. Similar to EC techniques, EDAs perform the optimization task by using binary encoding and selection operators over a set of potential solutions called population. The main difference regarding the classical EC techniques is that EDAs replace the crossover and mutation operators by building probabilistic models at each generation based on global statistical information of the best individuals.

By using these explicit probabilistic models, EDAs are able to solve optimization problems to cope with high level of epistasis. The principal advantages of EDAs over genetic algorithms are the absence of multiple parameters to be tuned and the expressiveness and transparency of the probabilistic model that guides the search process. EDAs have been proven to be better suited to some applications than GAs, while achieving competitive and robust results in the majority of tackled problems [24]. By the way, EDA is a discrete algorithm and PSO has been applied for continuous processes; although PSO has been applied in different works in discrete problems, its real potential is in the continuous domain [32]. PSO is similar to the genetic algorithm (GA) in the sense that these two evolutionary heuristics are population-based search methods by using a combination of deterministic and probabilistic rules. PSO has the same effectiveness as GA but with a significantly better computational efficiency [33]. Additional advantages of using EDAs are that they have the ability to adapt their operators, provide optimization practitioners with a Roadmap of how the problem was solved, use prior knowledge by injecting specific solutions, and require reduced memory and reduced computational times [29]. In this work, the Univariate Marginal Distribution Algorithm (UMDA) has been adopted as optimization strategy, because it is ideal for linear problems [23, 34, 35]. UMDA uses a binary codification for each possible solution, and it generates a probability vector **p** = (*p*_1_, *p*_2_, *p*_3_,…, *p*_*n*_)^*T*^, where *P*_*i*_ is the marginal probability of the *i*th bit of each individual, to be one or zero in the next generation. Then, UMDA tries to approximate the probability distribution of the individuals in *ℙ*. This can be defined as follows:

(4) $$\begin{array}{c} P\left(\;x\;\right)=\overset{n}{\underset{i=1}{\prod }}P\left(\;X_{i}=x_{i}\;\right), \end{array}$$

where *x* = (*x*_1_, *x*_2_,…, *x*_*n*_)^*T*^ is the binary value of the *i*th bit in the individual and *X*_*i*_ is the *i*th random value of the vector *X*. An objective function is needed to select a subset of best individuals. This function is used to determine the fitness of the current potential solution. A probability vector is computed from the subset of candidate solutions in order to generate a new population based on its distribution. This process is iteratively performed until a stop condition is achieved, and the best solution is chosen to be the individual with the best fitness value along the evolutionary process.

### 2.3. Proposed Method

This section describes the proposed method to detect parabolic shapes in images using the population-based method of UMDA along with the objective function to evaluate the fitness for each potential solution. The most representative steps of the proposed methodology for parabola detection are represented in Algorithm 1.

#### 2.3.1. Individual Representation

A parametric equation that describes a parabola is required to represent a potential solution for finding parabolic shapes in images. In the Cartesian coordinate system, a parabola can be described by using its general form as follows:

(5) $$\begin{array}{c} Ay^{2}+By+C=x, \end{array}$$

where *A*, *B*, and *C* represent unknown constant values, which can be determined using three independent pixels from the spatial image domain. Considering that the search space is a binary image, the coordinates (cols(*x*), rows(*y*)) of three random pixels are selected in order to form an individual for the population of the UMDA. For simplicity, the label *x* is used for cols(*x*) and *y* for rows(*y*).

The whole set of potential pixels in the input image are listed by their relative position to an origin, and they are labeled with an index ind = {1,2, 3,…, *N*}, where *N* is the total number of potential pixels. The coordinates (*x*_ind_, *y*_ind_) of the set {*i*, *j*, *k*} of pixels are used to compute the three constant values as follows:

(6) A=ykxj−xi+yjxi−xk+yixk−xjyi−yjyi−ykyj−yk,B=yk2xi−xj+yj2xk−xi+yi2xj−xkyi−yjyi−ykyj−yk,C=yjykyj−ykxi+ykyiyk−yixj+yiyjyi−yjxkyi−yjyi−ykyj−yk.

Finally, the coordinate values of the vertex and the aperture of the parabola can be computed by using the following:

(7) xvertex=−B2A,yvertex=C−B24A,4p=1A.

To illustrate the representation of a potential solution in an optimization model fitting process, Table 1 shows an example of an individual formed with the integer indexes {22,58,39} represented in base-2 numeral system that can be coded by 8-bits string. This individual is formed by concatenating the binary string indexes {*i*, *j*, *k*} of the three selected pixels in a single vector as it was used by Ayala-Ramirez et al. [6]. The number of genes for each individual directly depends on the number of potential pixels *N* in the spatial image domain.

Since the proposed method uses the indexes to form all the individuals, the UMDA method can easily eliminate unfeasible solutions by using a function to measure the quality of an individual.

#### 2.3.2. Fitness Function

To evaluate the fitness of an individual, a binary image *I*_VS_ (virtual shape) of the same size that the input image is generated. The value of all the pixels on the virtual image are set to zero, and this leads to a black color image. Then, the vertex and aperture 4*a* of the parabola represented by (5) are computed using the values *A*, *B*, and *C* obtained from the individual representation. Subsequently, the edge points of the parabola are computed and stored in *I*_VS_ by setting to 1 the corresponding pixels. Finally, the Hadamard product [36] between the binary form *f*_binary_ of the input image and the virtual image is calculated as follows:

(8) $$\begin{array}{c} Hd=f_{binary}⊙I_{VS}. \end{array}$$

**Figure 3:**
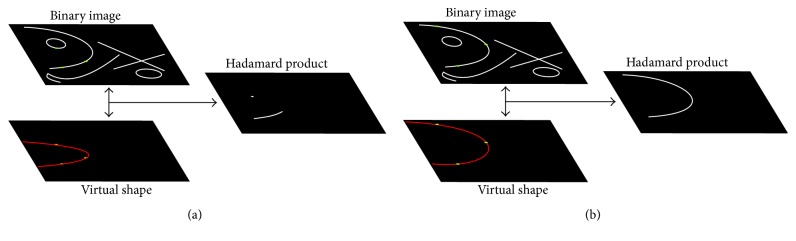
Hadamard product between virtual and input images.

The resulting image Hd contains only those pixels where *f*_binary_ and *I*_VS_ match.  Figure 3(a) illustrates an example with the three points taken from an individual to form a parabola in the virtual shape. It can be seen that the Hadamard product obtains partial information of the real parabola.  Figure 3(b) shows an example where the three points taken from the individual are part of a parabola that fits perfectly with the parabolic shape of the input image.

The fitness function used to assess the quality of potential solutions is the number of pixels resulting of the Hadamard product. Given that the binary image of the virtual shape is initialized with all pixels on black (intensity value of zero), with more matching pixels in Hd more white pixels will appear.

## 3. Results and Discussion

In this section, the proposed method for parabola detection is applied on synthetic images and medical images of the retina and human plantar arch. In order to assess the proposed method, it is compared with the Hough transform by applying the algorithm of Sanchez found in the MATLAB central [25] and by running the Hough transform for parabolic shapes algorithm provided by the MIPAV [26] software, that can be downloaded from the website [37]. However, considering that MIPAV and MATLAB parabola detection implementations are based on Hough transform, RANSAC algorithm [21] was implemented to analyze the performance of the proposed algorithm (cf. Tables 3 and 4).

The computational implementations are performed by using the MATLAB version 2013b, on a computer with an Intel Core i5, 4 GB of RAM, and 2.4 GHz processor. Moreover, computational experiments using UMDA were performed using 30 runs in order to perform a statistical analysis of the stochastic process applying the parameter values presented in Table 2.

These parameter settings were determined based on the solutions that give the best tradeoff between precision (lowest RMSE) and computational time using 30 trials to determine the best set of parameters. Moreover, different works were taken into account such as [23].

### 3.1. Application on Synthetic Images

**Figure 4:**
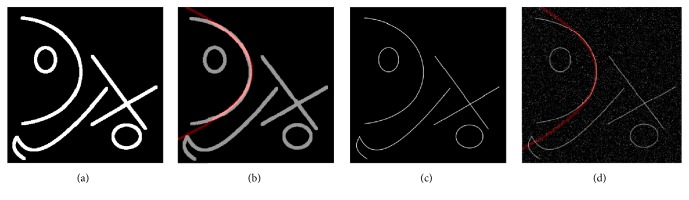
Parabola detection on synthetic image using the proposed method.

The first experiment was performed by using synthetic images like the one in Figure 4(a). This synthetic image was generated by drawing randomly located parametric objects such as lines, circles, and a parabola in order to evaluate the algorithm in a controlled way.

Table 3 presents a comparative analysis using Figure 4(a). The method presented in [25] shows the largest execution time, given that the result is the execution time per pixel and this image contains 125,899 pixels. Hence, this method depends entirely on the number of potential pixels. The proposed method performs a reduction of 93.61% of the execution time achieved with the MIPAV software, given that the UMDA method has 10 individuals and taking into account the mean of the number of iterations, only 195 function evaluations are required to obtain the best result as it is shown in Figure 4(b).

To ensure that the algorithm is robust and results are consistent with different input conditions, the complexity of the image was increased by adding “salt and pepper” noise. The test was performed by obtaining the skeleton of Figure 4(a) and adding a 10% of “salt and pepper” noise, as it is shown in Figure 4(d). In Table 4, a comparison of the three methods using Figure 4(d) is illustrated. Since the method in [25] works over a single pixel, the result is consistent with Table 3. The proposed method achieves a reduction of 96.14% in comparison with the MIPAV software. Considering that the amount of pixels is lower than the binary image, the number of iterations is congruent with the reduction in the execution time. In this test, the average number of function evaluations to obtain the best result using UMDA was 173 (Figure 4(d)).

In order to quantify the fitness of detected parabolas, ten synthetic images with parabolas were generated using (5) with random values of *A*, *B*, and *C*.* Salt and pepper* noise in the regular range of [0, 25%] was added to the images giving a total of 260 test images. Normalized root-mean-square deviation (RMSD) or root-mean-square error (RMSE) was computed to compare original parabolas to the one detected by the proposed algorithm, according to the following:

(9) $$\begin{array}{c} RMSD=\sqrt{\frac{1}{n}\overset{n}{\underset{t=1}{\sum }}{\left(x_{real}-x_{detected}\right)}^{2}}. \end{array}$$

To obtain a true normalized version, (9) can be divided by max(*x*_real_), mean(*x*_real_), or Cardinality(*x*_real_ = = *x*). In our experiments, the Cardinality was used to prioritize the fitting curves having maximum number of matched points.

**Figure 5:**
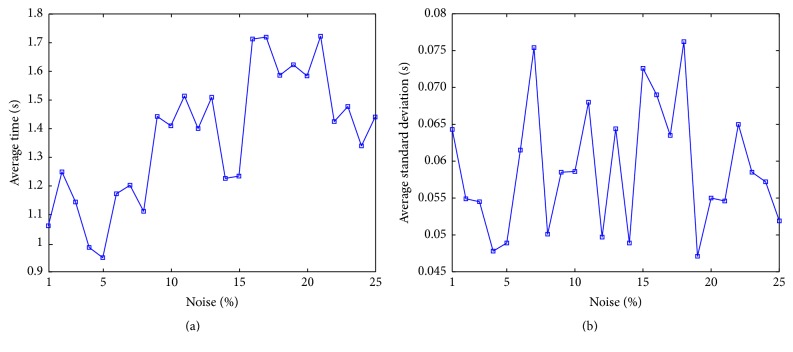
Average computational time and standard deviation (in seconds) over 30 runs of UMDA per percentage noise for the skeleton image (Figure 4(c)).

A test by adding “salt and pepper” noise over the range [1,25] percent to Figure 4(c) (skeleton image) was carried out in order to evaluate the performance of the proposed method in terms of computational time with different amounts of noise.  Figure 5(a) shows the average execution time of the obtained results. Since the image has fewer potential pixels than the binary image, the execution time with 1% of noise was low in comparison with previous results. Considering that the UMDA method is a stochastic algorithm, its performance is not shown as a straight line; however, the mean time tends to increase when the amount of noise is also increased.  Figure 5(b) shows the standard deviation of the performed tests; since the graph tends to a constant mean over all the percentages of noise, it can be assumed that the algorithm is stable and robust in the presence of noise over the time (i.e., noise may affect quality results). This time stability is originated by the normalized RMSD metric, because more noisy pixels are considered as part of the detected parabola while the global error is also increasing. However, it is observed that most methods (RANSAC, MIPAV, and Hough transform) fail to detect correctly the parabola parameters on images having more than 25% of* salt and pepper* noise, and the proposed method could detect parabolas on synthetic and real images contaminated until 30%.

**Figure 6:**
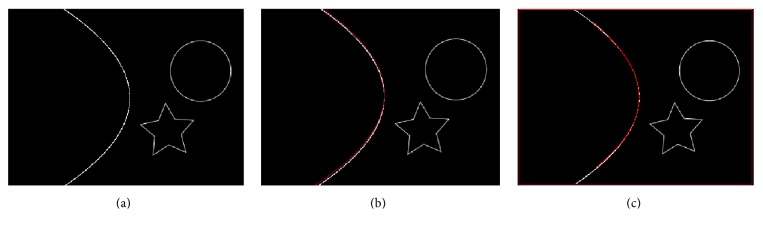
Parabola detection performed by the proposed approach and MIPAV software.

**Figure 7:**
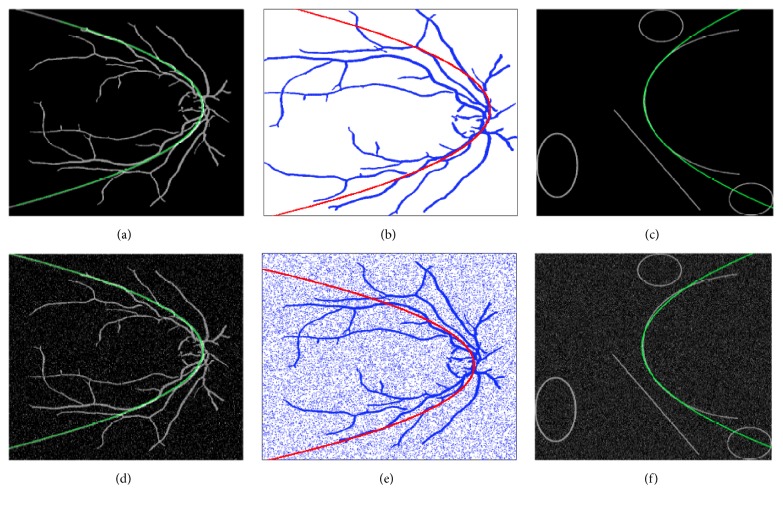
Comparative images between RANSAC and the proposed method (noise added). (a) Retinal image process by the proposed algorithm, (b) retinal image processed by RANSAC, (c) synthetic image processed by the proposed method, and (d, e, f) images from (a, b, c) with 15% of* salt and pepper* noise, respectively.

Figure 6(a) is a synthetic image for fitness testing, and Figures 6(b) and 6(c) present detected parabolas using our proposed algorithm and MIPAV software, respectively. RMSD values for both times of individual detection are 0.0026 and 0.0089, demonstrating the validity of our method. In Figure 7, comparative results between the proposed method and RANSAC are depicted, and Table 5 shows the normalized errors in parabola detection on original images and with 15% of noise. The fast and stable performance of the proposed algorithm compared to RANSAC is noticed; besides, RANSAC has many parameters to tune and is computationally expensive and there is no warranty for reproducing the same results in a new evaluation.

### 3.2. Retinal Fundus Images

The detection of parametric objects has been applied in different areas of engineering. In medical imaging, the detection of parabolas in retinal fundus images has a particular importance, since the form of the retinal vessels can be approximated to the parabola parametric form. In the previous work, the standard model, the Hough transform, has been used to find the parabola that fits the best on the retinal images. For instance, in the work reported by Oloumi et al. [10, 11], the form of the retina vessels is approximated to a parabola with the aim of monitoring the measurements of the openness of the major temporal arcade (MTA). This study facilitates the quantitative analysis of the MTA and overcomes the limitations associated with manual analysis. Another application is reported by Yu et al. [38], where the retinal images are used to find the vertex of all the vessels; this vertex is known as fovea [39]. This study is significant because the position of the vertex can give the grade of diabetic retinopathy.

**Figure 8:**
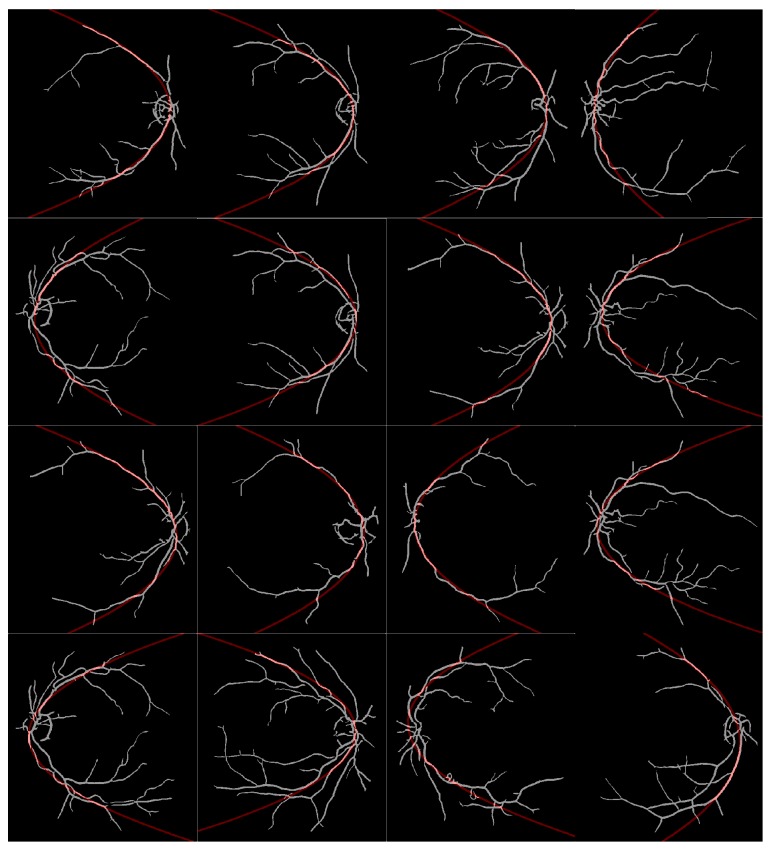
Results of parabola detection using the proposed method over a subset of retinal fundus images from the DRIVE database.

In the tests reported in the present work, the proposed method was used to approximate the retinal vessels to a parabola.  Figure 8 shows the results obtained, where the best fit for the parabola in the image is shown in red color. As in the work reported by Yu et al. [38], the public database DRIVE [40] that contains a set of 20 retinal fundus images was used. Images were preprocessed by applying Gaussian matched filters for vessel detection.

Table 6 presents the statistical analysis of the results obtained by applying the proposed and comparative methods to the DRIVE database. The method presented in [25] remains constant with the execution time per pixel; then, this execution time for this algorithm is the largest time. Since the proposed method accomplishes the best result with only 165 function evaluations, it is possible to achieve a reduction in terms of computational time of 89% in comparison with the one performed on the MIPAV software.

Given that the skeleton of an image passes through the center section of a set of pixels, if the proposed algorithm is applied to the skeleton of the retinal images, it is then expected for the algorithm to achieve a lower execution time than in the binary image. In Table 7, the statistics of the execution time for the skeleton images are shown. The results are consistent with those obtained for the synthetic image, and as it can be seen, the proposed method clearly outperforms the execution time of HT algorithm from Sanchez and the MIPAV software.

Since the proposed method takes three pixels to compute a parabola, it can be assumed that the number of iterations to achieve the best result is not related to the size of the image. This assumption is validated with the results shown in Tables 6 and 7, where the average number of iterations for both the binary image and the skeleton image is less than 30.

### 3.3. Plantar Arch Images

The form of the plantar arch in humans can conduct to several conditions if the contact area with the floor, given by the plantar arch, is not of the correct size. In clinical practice, the procedure for providing a diagnosis of flatfoot degree is by visual inspection of the specialist. Therefore, the proposed method in this work can be applied to plantar arch images to approximate the heel and the plantar arch to parabolas; hence, these parameters could be used to assist a medical diagnostic. The image database used in this test was created by the authors and approved by a specialist (Dr. Carlos Reséndiz Ramírez). This database is composed of 80 images of size 285 × 707 pixels in RGB color space of left and right foot collected from different patients. Images were first preprocessed, to reduce the amount of data that was not useful for this study, as described below.

Let *I* be a grayscale image of the foot to be analyzed, with *M* and *N* as the number of pixels for the width and height, respectively; then,

(10) $$\begin{array}{c} I=f\left(\;x,y\;\right),\;x\in \left[0,M-1\right],\;\;y\in \left[0,N-1\right]. \end{array}$$

The first step of the method consists in smoothing the image by applying a mean filter, that is, convolving the image with a filter mask:

(11) $$\begin{array}{c} I_{smooth}=I⋆f_{smooth}, \end{array}$$

where *f*_smooth_ is defined by

(12) $$\begin{array}{c} f_{smooth}=\left[\begin{array}{ccc} \frac{1}{9} & \frac{1}{9} & \frac{1}{9}\\ \frac{1}{9} & \frac{1}{9} & \frac{1}{9}\\ \frac{1}{9} & \frac{1}{9} & \frac{1}{9} \end{array}\right]. \end{array}$$

This filter removes part of the spurious pixels, improving the probability of finding the curves of interest. After the smoothing step, gradients are computed on the image in order to find the edges of the footprint. Canny edge detection is the algorithm used to perform this step. The resulting contour image *f*_edge_ is then dilated (13) by using a Structuring Element (SE) of radius 3, where the dilation operation closes slightly open contours and makes borders thicker.

(13) $$\begin{array}{c} f_{binary}=f_{edge}⊕SE. \end{array}$$

The *f*_binary_ image is the result of the preprocessing stage in the proposed method. This image is referred to as the* edge image*.

In order to address the plantar arch issue, the foot image was divided into three sections from the fingers to the heel, where each section contains 33.33% of the image. The sections of interest for this study are only those two that include the plantar arch and the heel.

**Figure 9:**
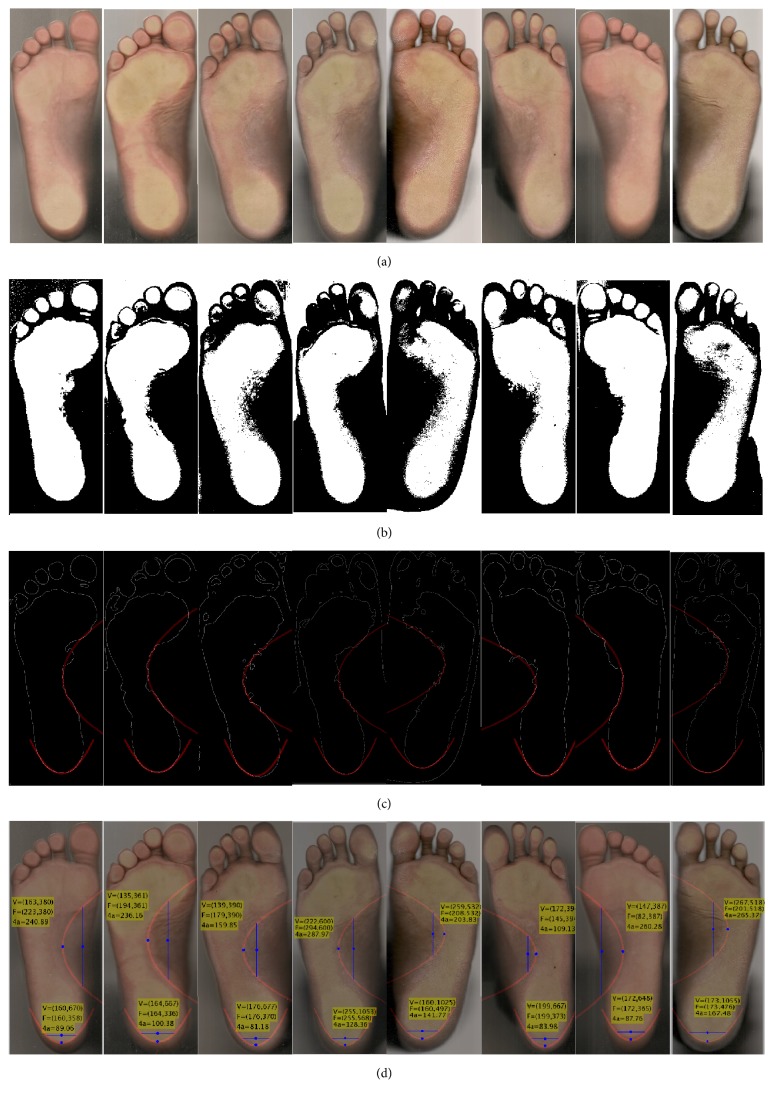
(a) Subset of human plantar arch images. (b) Preprocessing step applied to the images in (a). (c) Results of parabola detection (plantar arch and heel) over the edge images. (d) Quantitative analysis of the detected parabolas from the images in (a).

Table 8 presents a comparative analysis of the execution time of the algorithms over the database of the human plantar arch images. As it can be observed, the method developed in [25] maintains the average time per pixel, and so, it takes the largest execution time. The proposed method performs a reduction of 89.96% of the execution time achieved on the MIPAV software; this is because the algorithm requires only an average of 135 function evaluations to obtain the best result.  Figure 9 shows a subset of human plantar arch images, where the heel and plantar arch were parameterized with the proposed method.

In addition, the comparative results in Table 8 show that the proposed method outperforms the HT algorithm from Sanchez and the algorithm from the MIPAV software in terms of computational time.  Figure 9 presents the results of the proposed method in different plantar arch images, where the rows, from (a) to (d), show the real images, the binary images, the edge images with the detected parabolas, and the output of the proposed method.

**Figure 10:**
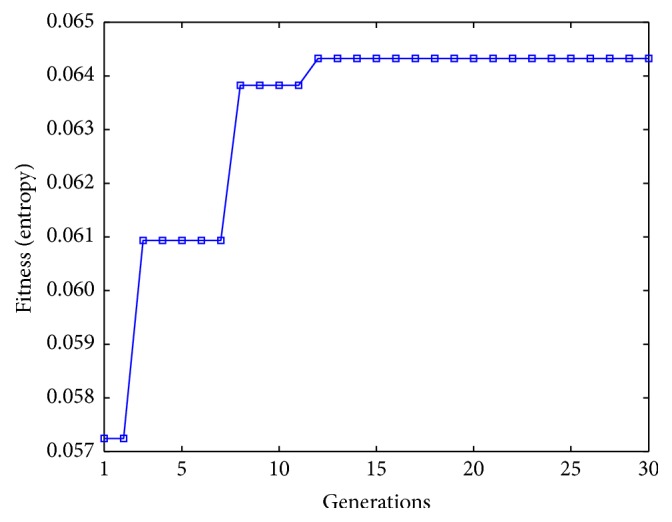
Evolution of the average fitness through generations using the database of human plantar arch images.

Finally, Figure 10 shows the average fitness value through the generations of UMDA over the 30 runs of the proposed method. This graph represents the iterations that were required by the UMDA method to find the two parabolas in the image.

## 4. Conclusion

In this paper, a new method based on the Estimation of Distribution Algorithms (EDAs) has been proposed to detect parabolic shapes. The method computes the constant values of the generic parabola equation by selecting three random pixels from the input image. The proposed method was evaluated in terms of computational time and compared with freely available implementations of the parabola Hough transform. According to experimental results, the average time of the proposed method is significantly better (0.0036 seconds) compared to those obtained by the Hough transform, since this method takes an average of 6.14 seconds to compute the accumulator for one single boundary pixel. In addition, experimental results have also shown that the proposed method outperforms the two comparative methods in terms of execution time saving 93.61% on synthetic images and 89% on the medical images of retinal fundus and human plantar arch.

## Figures and Tables

**Algorithm 1:**
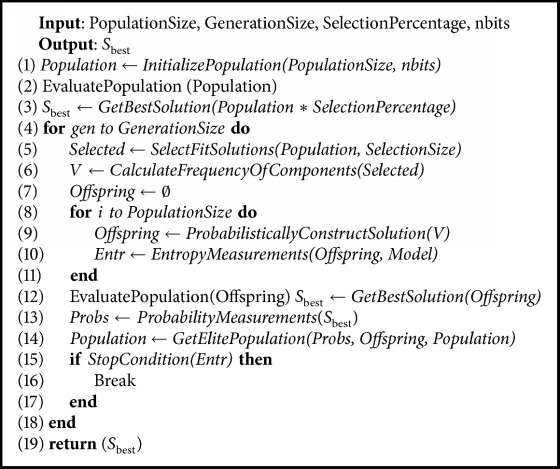
Fast parabola detection method by UMDA.

**Table 1:** Example of an individual with 3 indexes where each index represents an 8-bit pixel position.

Individual																							
Index *i*								Index *j*								Index *k*							
0	0	0	1	0	1	1	0	0	0	1	1	1	0	1	0	0	0	1	0	0	1	1	1

**Table 2:** UMDA parameters for all the computational experiments.

Parameter	Value
Number of individuals	10
Selection rate	0.6
Maximum number of generations	30

**Table 3:** Comparative analysis of execution time using the binary image (Figure 4(a)).

Method	Execution time (s)		Number of iterations	
Proposed method	Minimum	1.7141	Minimum	15
	Maximum	3.5928	Maximum	28
	Mean	2.3756	Mean	19.55
	Median	2.1934	Median	19

Hough transform [25]	8.8205 per pixel		—	

MIPAV [26]	37.68		—	

RANSAC [27]	16.35		5000	

**Table 4:** Comparative analysis of execution time using the skeleton of the binary image (Figure 4(c)).

Method	Execution time (s)		Number of iterations	
Proposed method	Minimum	0.2820	Minimum	13
	Maximum	3.2988	Maximum	29
	Mean	1.4163	Mean	17.33
	Median	1.4326	Median	18

Hough transform [25]	8.8205 per pixel		—	

MIPAV [26]	36.47		—	

RANSAC [27]	14.86		5000	

**Table 5:** Comparative RMSE for synthetic and real images shown in Figure 7.

Algorithm	Parameters (*h*, *k*) *P*	Time (s)	RMSD	uRMSD_ _^*∗*^	Matched points
Proposed method	(468,276) −40.92	4.464	9.330	64.230	1439
RANSAC [27]	(468,301) −44.55	12.993	8.453	211.137	1625
Proposed method	(502,512) 135.2	15.490	13.895	426.277	2752
Proposed method	(464,278) −43.74	4.022	9.641	77.995	1939
RANSAC [27]	(431,259) −54.18	27.099	13.932	340.910	1258
Proposed method	(508,522) 130.6	4.357	9.712	182.037	4178

^*∗*^Unnormalized RMSD/RMSE.

**Table 6:** Comparative analysis of the average execution time using the DRIVE database of retinal fundus images.

Method	Execution time (s)		Number of iterations	
Proposed method	Minimum	1.2280	Minimum	5
	Maximum	6.3285	Maximum	22
	Mean	4.5838	Mean	16.28
	Median	5.1588	Median	19

Hough transform [25]	8.8205 per pixel		—	

MIPAV [26]	43.65		—	

RANSAC [21]	16.66		5000	

**Table 7:** Comparative analysis of execution time using the skeleton of the retinal fundus images (DRIVE database).

Method	Execution time (s)		Number of iterations	
Proposed method	Minimum	0.9550	Minimum	4
	Maximum	5.7105	Maximum	20
	Mean	3.3706	Mean	12
	Median	2.7343	Median	10

Hough transform [25]	8.8205 per pixel		—	

MIPAV [26]	41.87		—	

RANSAC [21]	12.99		5000	

**Table 8:** Comparative analysis of execution time using the database of human plantar arch images.

Method	Execution time (s)		Number of iterations	
Proposed method	Minimum	4.2118	Minimum	3
	Maximum	7.1688	Maximum	26
	Mean	6.18	Mean	13.51
	Median	6.40	Median	14.15

Hough transform [25]	8.8205 per pixel		—	

MIPAV [26]	63.78		—	

RANSAC [21]	22.54		5000	

## References

[1] Duda R. O., Hart P. E. (1972). Use of the hough transformation to detect lines and curves in pictures. *Communications of the ACM*.

[2] Illingworth J., Kittler J. (1988). A survey of the hough transform. *Computer Vision, Graphics and Image Processing*.

[3] Atherton T. J., Kerbyson D. J. Using phase to represent radius in the coherent circle Hough transform.

[4] Yuen H., Princen J., Illingworth J., Kittler J. (1990). Comparative study of Hough Transform methods for circle finding. *Image and Vision Computing*.

[5] Mukhopadhyay P., Chaudhuri B. B. (2015). A survey of hough transform. *Pattern Recognition*.

[6] Ayala-Ramirez V., Garcia-Capulin C. H., Perez-Garcia A., Sanchez-Yanez R. E. (2006). Circle detection on images using genetic algorithms. *Pattern Recognition Letters*.

[7] Cuevas E., Ortega-Sánchez N., Zaldivar D., Pérez-Cisneros M. (2012). Circle detection by harmony search optimization. *Journal of Intelligent and Robotic Systems: Theory and Applications*.

[8] Maalmi K., El-Ouaazizi A., Benslimane R., Lew Yan L. F. C., Voon A., Gorria P. Crack defect detection and localization using genetic-based inverse voting hough transform.

[9] Oloumi F., Rangayyan R. M. (2009). Detection of the temporal arcade in fundus images of the retina using the Hough transform. *Conference proceedings IEEE Engineering in Medicine and Biology Society*.

[10] Oloumi F., Rangayyan R. M., Ells A. L. Computer-aided diagnosis of proliferative diabetic retinopathy.

[11] Oloumi F., Rangayyan R. M., Ells A. L. (2012). Parabolic modeling of the major temporal arcade in retinal fundus images. *IEEE Transactions on Instrumentation and Measurement*.

[12] Cai P., Wang C. (2015). An eyelid detection algorithm for the iris recognition. *International Journal of Security and its Applications*.

[13] Nabi F., Yousefi H., Soltanian-Zadeh H. Segmentation of major temporal arcade in angiography images of retina using generalized hough transform and graph analysis.

[14] Lee G., Pollo F. E. (2001). Technology overview: the gait analysis laboratory. *Journal of Clinical Engineering*.

[15] Frykberg R. G., Lavery L. A., Pham H., Harvey C., Harkless L., Veves A. (1998). Role of neuropathy and high foot pressures in diabetic foot ulceration. *Diabetes Care*.

[16] Kulkarni P. S., Kulkarni V. B. Human footprint classification using image parameters.

[17] Chu W. C., Lee S. H., Chu W., Wang T.-J., Lee M.-C. (1995). The use of arch index to characterize arch height: a digital image processing approach. *IEEE Transactions on Biomedical Engineering*.

[18] Zheng S., Huang K., Tan T., Tao D. (2012). A cascade fusion scheme for gait and cumulative foot pressure image recognition. *Pattern Recognition*.

[19] Salehin M., Zheng L., Gao J. Conics detection method based on pascal's theorem.

[20] Merazi-Meksen T., Boudraa M., Boudraa B. (2014). Mathematical morphology for TOFD image analysis and automatic crack detection. *Ultrasonics*.

[21] Niedfeldt P. C., Beard R. W. (2013). Recursive ransac: multiple signal estimation with outliers. *IFAC Proceedings Volumes*.

[22] Hassner T., Assif L., Wolf L. (2014). When standard RANSAC is not enough: cross-media visual matching with hypothesis relevancy. *Machine Vision and Applications*.

[23] Cruz-Aceves I., Hernandez-Aguirre A., Valdez S. I. (2016). On the performance of nature inspired algorithms for the automatic segmentation of coronary arteries using Gaussian matched filters. *Applied Soft Computing Journal*.

[24] Armañanzas R., Inza I., Santana R. (2008). A review of estimation of distribution algorithms in bioinformatics. *BioData Mining*.

[25] Sanchez C. (2007). *Parabola Detection Using Hough Transform*.

[26] McAuliffe M. J., Lalonde F. M., McGarry D., Gandler W., Csaky K., Trus B. L. Medical image processing, analysis and visualization in clinical research.

[27] Fischler M. A., Bolles R. C. (1981). Random sample consensus: a paradigm for model fitting with applications to image analysis and automated cartography. *Communications of the Association for Computing Machinery*.

[28] Ballard D. H. (1981). Generalizing the Hough transform to detect arbitrary shapes. *Pattern Recognition*.

[29] Hauschild M., Pelikan M. (2011). An introduction and survey of estimation of distribution algorithms. *Swarm and Evolutionary Computation*.

[30] Larrañaga P., Lozano J. A. (2002). *Estimation of Distribution Algorithms: A New Tool for Evolutionary Computation*.

[31] Larrañaga P. (2002). A review on estimation of distribution algorithms. *Estimation of Distribution Algorithms*.

[32] Kennedy J., Eberhart R. Particle swarm optimization.

[33] Hassan R., Cohanim B., de Weck O., Venter G. A comparison of particle swarm optimization and the genetic algorithm.

[34] Pelikan M., Goldberg D. E., Lobo F. G. (2002). A survey of optimization by building and using probabilistic models. *Computational Optimization and Applications. An International Journal*.

[35] Mühlenbein H., Paaß G. (1996). From recombination of genes to the estimation of distributions I. Binary parameters. *Parallel Problem Solving from Nature—PPSN IV*.

[36] Million E. (2007). *The Hadamard Product*.

[37] National Institutes of Health Center for Information Technology (2015). *Medical Image Processing, Analysis and Visualization*.

[38] Yu C.-Y., Liu C.-C., Yu S.-S. (2014). A fovea localization scheme using vessel origin-based parabolic model. *Algorithms*.

[39] Gegundez-Arias M. E., Marin D., Bravo J. M., Suero A. (2013). Locating the fovea center position in digital fundus images using thresholding and feature extraction techniques. *Computerized Medical Imaging and Graphics*.

[40] Staal J., Abràmoff M. D., Niemeijer M., Viergever M. A., Van Ginneken B. (2004). Ridge-based vessel segmentation in color images of the retina. *IEEE Transactions on Medical Imaging*.

